# A biomathematical model of immune response and barrier function in mice with pneumococcal lung infection

**DOI:** 10.1371/journal.pone.0243147

**Published:** 2020-12-03

**Authors:** Sibylle Schirm, Peter Ahnert, Sarah Berger, Geraldine Nouailles, Sandra-Maria Wienhold, Holger Müller-Redetzky, Norbert Suttorp, Markus Loeffler, Martin Witzenrath, Markus Scholz

**Affiliations:** 1 Institute for Medical Informatics, Statistics and Epidemiology, University of Leipzig, Leipzig, Germany; 2 Division of Pulmonary Inflammation, Charité - Universitätsmedizin Berlin, corporate member of Freie Universität Berlin, Humboldt-Universität zu Berlin, and Berlin Institute of Health, Berlin, Germany; 3 Department of Infectious Diseases and Respiratory Medicine, Charité - Universitätsmedizin Berlin, corporate member of Freie Universität Berlin, Humboldt-Universität zu Berlin, and Berlin Institute of Health, Berlin, Germany; 4 LIFE Research Center of Civilization Diseases, University of Leipzig, Leipzig, Germany; Monash University, AUSTRALIA

## Abstract

Pneumonia is one of the leading causes of death worldwide. The course of the disease is often highly dynamic with unforeseen critical deterioration within hours in a relevant proportion of patients. Besides antibiotic treatment, novel adjunctive therapies are under development. Their additive value needs to be explored in preclinical and clinical studies and corresponding therapy schedules require optimization prior to introduction into clinical practice. Biomathematical modeling of the underlying disease and therapy processes might be a useful aid to support these processes. We here propose a biomathematical model of murine immune response during infection with *Streptococcus pneumoniae* aiming at predicting the outcome of different treatment schedules. The model consists of a number of non-linear ordinary differential equations describing the dynamics and interactions of the pulmonal pneumococcal population and relevant cells of the innate immune response, namely alveolar- and inflammatory macrophages and neutrophils. The cytokines IL-6 and IL-10 and the chemokines CCL2, CXCL1 and CXCL5 are considered as major mediators of the immune response. We also model the invasion of peripheral blood monocytes, their differentiation into macrophages and bacterial penetration through the epithelial barrier causing blood stream infections. We impose therapy effects on this system by modelling antibiotic therapy and treatment with the novel C5a-inactivator NOX-D19. All equations are derived by translating known biological mechanisms into equations and assuming appropriate response kinetics. Unknown model parameters were determined by fitting the predictions of the model to time series data derived from mice experiments with close-meshed time series of state parameters. Parameter fittings resulted in a good agreement of model and data for the experimental scenarios. The model can be used to predict the performance of alternative schedules of combined antibiotic and NOX-D19 treatment. We conclude that we established a comprehensive biomathematical model of pneumococcal lung infection, immune response and barrier function in mice allowing simulations of new treatment schedules. We aim to validate the model on the basis of further experimental data. We also plan the inclusion of further novel therapy principles and the translation of the model to the human situation in the near future.

## Introduction

*Streptococcus pneumoniae* infections are life-threatening especially in children or elderly patients [1]. The disease can rapidly deteriorate within hours requiring immediate intensive care. However, such fulminant disease courses only affect a part of the patient population and it is so far difficult to predict which patients are at high risk.

Besides antibiotic treatment, new adjuvant therapy options are under development. For example the recently developed drug NOX-D19 spiegelmer targets C5a, a component of the complement system responsible for several proinflammatory effects including tissue injury [2]. It was observed in [3] that NOX-D19 reduces lung permeability in mice. Therefore, there is some hope that anti-C5a treatment might help stabilizing barrier function, reducing bacteremia and improving outcome [4]. So far, its relative contribution in combination with antibiotic treatment is not explored.

We recently proposed a biomathematical model of *Streptococcus pneumoniae* infection in mice [5] considering major cellular players of the innate immune response. This model was designed to simulate different schedules of antibiotic treatment. We here propose a significant extension of this work by modelling the barrier function in more detail. We also extend the network of explicitly modelled cytokines and chemokines and propose mechanistic assumptions for the action of NOX-D19 on the system with the aim to simulate combined therapies of antibiotics and NOX-D19. For our modelling, we translated biological knowledge and hypotheses about the key players of the innate immune response and therapy effects into mathematical equations and compare simulation results with available data. For this purpose, we can rely on an extended data base including early time points after infection and NOX-D19 or antibiotic treatment.

## Methods

### General structure of the model

We revise and extend our recently published model of pneumococcal infection in mice [5] which is based on a model proposed by Smith et al. [6]. Our former model described the dynamics of pneumococci, neutrophils, macrophages, destruction of the epithelial barrier and IL-6 in lung-infected mice using ordinary differential equations. Effects of the antibiotic drugs Moxifloxacin or Ampicillin were included.

**Fig 1:**
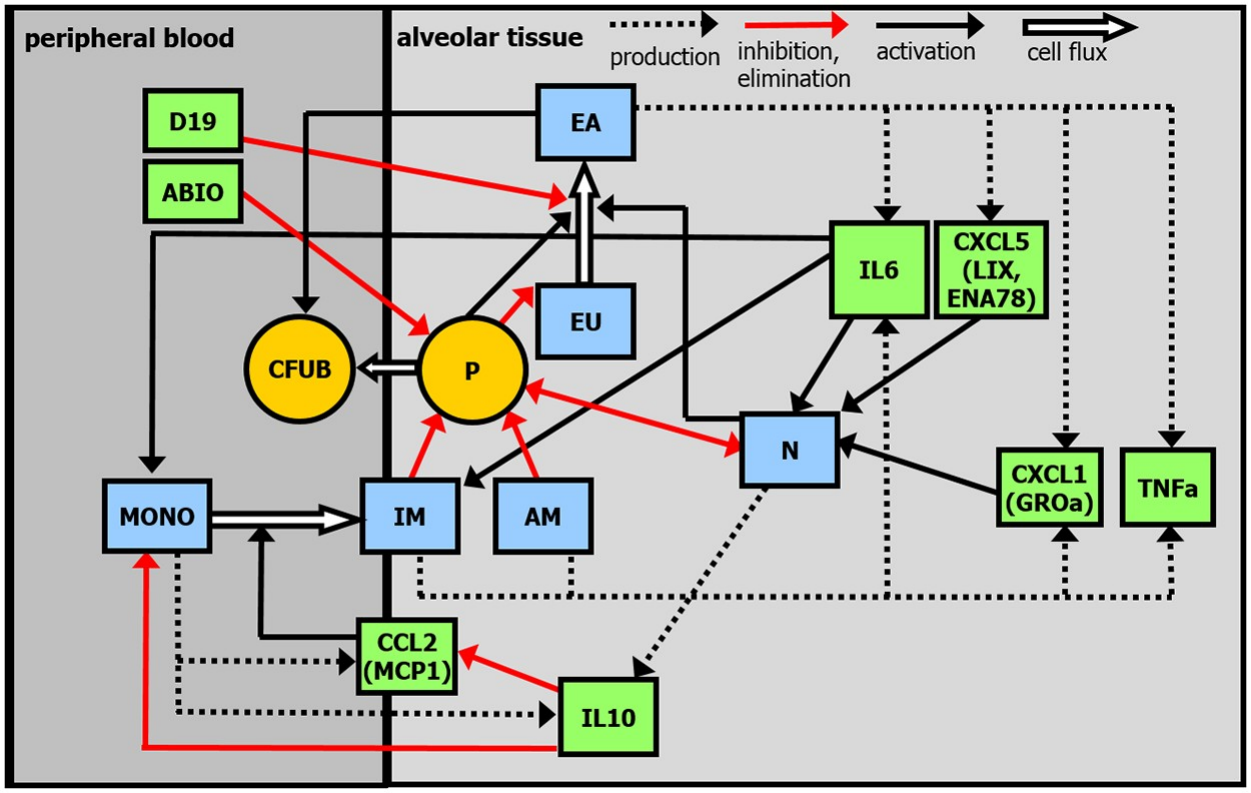
Structure of the model. Compartments: pneumococci in lungs (P), pneumococci in peripheral blood (CFUB), unaffected epithelial/endothelial cells (EU), affected epithelial/endothelial cells (EA), alveolar macrophages (AM), inflammatory macrophages (IM), neutrophils (N), monocytes in peripheral blood (MONO), cytokine / chemokine network comprising IL-6, IL-10, CCL2, CXCL1, CXCL5 and TNF*α*. ABIO denotes the antibiotic therapy. D19 represents the effect of NOX-D19 treatment.

Based on new experimental data, we aim at refining this model. First, we now distinguish between alveolar macrophages and inflammatory macrophages, which was impossible on the basis of earlier data. Second, we explicitly model the migration of monocytes from peripheral blood to the alveolar space and their differentiation into inflammatory macrophages. This process is mainly regulated by the cytokines IL-6 and IL-10 and the chemokine CCL2 (MCP-1). Epithelial barrier function is also modeled in more detail to understand, e.g. barrier breakdown and effects of NOX-D19 treatment including their impact on the migration of pneumococci into blood stream. Finally, neutrophil attraction by the chemokines CXCL1 (GROa) and CXCL5 (LIX, ENA78) is considered. This requires extension of our cytokine / chemokine network to be modelled. All model equations are based on biologically motivated hypotheses translated into differential equations. We justify these assumptions and present derived equations in the following. Major compartments and regulations of our model are presented in Fig 1.

### Model equations and assumptions

#### Compartment P: Pneumococci

Healthy mice are infected with pneumococci transnasally. This is modelled in analogy to our former model [5].

$$\begin{array}{c} \text{PneuStart}=\text{EffInf}\sum_{i=1}N\frac{{\text{dose}\_\text{pneu}}_{i}}{t_{\text{Pneu}}}(\text{Hv}\left(t-\tilde{t_{i}}\right)-\text{Hv}\left(t-\tilde{t_{i}}-t_{\text{Pneu}}\right)) \end{array}$$ (1)

where Hv is the Heaviside-function $\text{Hv}=\{\begin{array}{ccc} 0 & : & x<0\\ 1 & : & x\geq 0 \end{array}$, $\tilde{t_{i}}$ are the time points at which pneumococci at dose dose_pneu_i_ are administered in time *t*_Pneu_. Here we only consider single infections, i.e. *N* = 1, $\tilde{t_{1}}=0$, dose_pneu_1_ = 5 ⋅ 10^6^ and *t*_Pneu_ = 1min. The infection function is multiplied by a seeding efficacy EffInf = 0.001 translating applied amount of bacteria to those in 1*μl* of broncho-alveolar lavage (BAL) directly after infection. Measurements two hours after infection are in between 320-2560 CFU/*μl*. The factor EffInf indirectly accounts for physical barriers of entry for pathogens, which are assumed to be equal for all animals, and therefore, have no impact on infection dynamics.

Pneumococci population in the alveolar space initially grows with rate *k*_*P*_ until a saturation *P*_max_ is reached. Bacteria were eliminated by several lines of innate immune defense comprising alveolar macrophages, neutrophils, and finally, inflammatory macrophages [6]. The defense mechanisms are assumed to be saturated by a Michaelis-Menten kinetic representing the limited killing capacity of immune cells. Pneumococci pass the epithelial/endothelial barrier and enter the blood-stream with rate *k*_CFUB_P_ and in dependence on bacterial load and permeability of the barrier described by the amount of affected epithelial cells (EA, see below). Antibiotic treatment imposes a time dependent loss rate *A*(*t*) on the pneumococci population. Summarizing these assumptions results in the following equation.

$$\begin{array}{ccc} \frac{dP}{dt} & = & \text{PneuStart}+k_{P}\cdot P\left(t\right)\cdot (1-\frac{P(t)}{P_{\text{max}}})\\ & & -(k_{P\_\text{AM}}\cdot AM_{0}+k_{P\_N}\cdot N\left(t\right)+k_{P\_\text{IM}}\cdot IM\left(t\right))\cdot \frac{P(t)}{n+P(t)}\\ & & -k_{\text{CFUB}\_P}\cdot P\left(t\right)\cdot EA(t)-A\left(t\right)\cdot P\left(t\right) \end{array}$$ (2)

The time dependent antibiotic effect *A*(*t*) is modelled by injection functions of antibiotics and a resulting (delayed) effect on pneumococci. First, injections of antibiotic drugs ABIO^inj^(*t*) are modelled by a sum of pulse functions which can be written as

$$\begin{array}{cc} {\text{ABIO}}^{\text{inj}}\left(t\right)= & \;\frac{{\text{dose}}_{\text{ABIO}}\cdot k_{\text{ABIO}}}{t_{\text{ABIO}}}\cdot \sum_{i=1}^{N}(\text{Hv}\left(t-\tilde{t_{i}}\right)-\text{Hv}\left(t-\tilde{t_{i}}-t_{\text{ABIO}}\right)) \end{array}$$ (3)

where $\tilde{t_{i}}$ are the time points at which antibiotics at a dose of “dose_ABIO_” were administered. *k*_ABIO_ describes the antibiotic effect of the administered drug, i.e. its toxicity to pneumococci. The duration of the injections (*t*_ABIO_) is set to 0.1 hour. The applied dose is normalized to this injection time by the factor 1/*t*_ABIO_.

In analogy to [5], we assume that the antibiotic effect after injection is delayed. This is modeled by two delay compartments with first order transitions, where $C_{\text{ABIO}}^{\left(i\right)}\left(t\right)$ is the concentration of the antibiotic drug in the delay compartment *i*:

$$\begin{array}{c} \frac{dC_{\text{ABIO}}^{\left(i\right)}\left(t\right)}{dt}=C_{\text{ABIO}\_\text{out}}^{\left(i-1\right)}\left(t\right)-k_{\text{ABIO}}^{\text{Delay}}\cdot C_{\text{ABIO}}^{\left(i\right)}\left(t\right)\;i=1,2 \end{array}$$ (4)

with the settings

$$\begin{array}{c} C_{\text{ABIO}\_\text{out}}^{\left(0\right)}\left(t\right)={\text{ABIO}}^{\text{inj}}\left(t\right) \end{array}$$

and

$$\begin{array}{c} C_{\text{ABIO}\_\text{out}}^{\left(i\right)}\left(t\right)=k_{\text{ABIO}}^{\text{Delay}}\cdot C_{\text{ABIO}}^{\left(i\right)}\left(t\right)\;i=1,2 \end{array}$$

**Fig 2:**
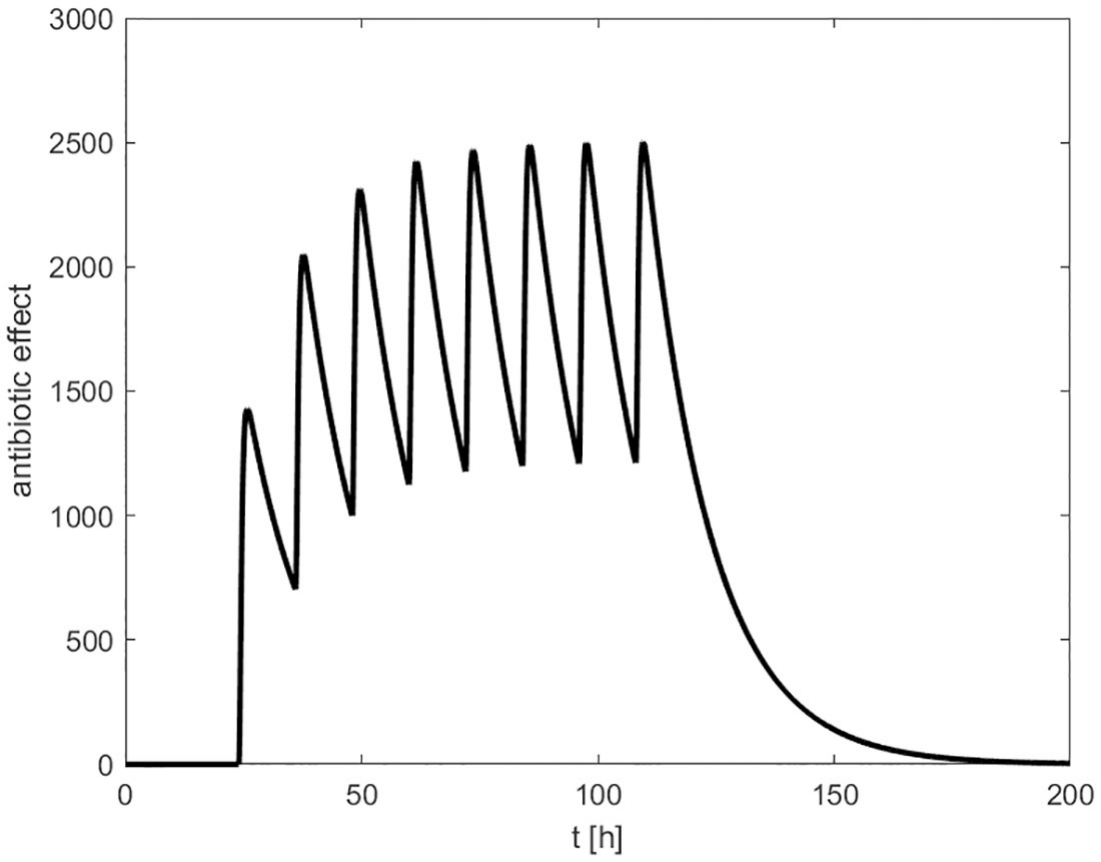
Antibiotic therapy. In this example, the antibiotic effect *A*(*t*) of repetitive drug administrations in time intervals of 12 hours is shown. Therapy starts 24 hours after infection and stops after 8 injections.

$C_{\text{ABIO}\_\text{out}}^{\left(2\right)}\left(t\right)$ enters the compartment of antibiotic effect on pneumococci:

$$\begin{array}{cc} \frac{dA(t)}{dt} & =C_{\text{ABIO}\_\text{out}}^{\left(2\right)}\left(t\right)-d_{\text{ABIO}}\cdot A\left(t\right) \end{array}$$ (5)

where *A*(*t*) corresponds to the antibiotic effect and *d*_ABIO_ is the waning rate. The antibiotic effect *A*(*t*) of repetitive applications is illustrated in Fig 2.

#### Compartments EU and EA: Unaffected and affected epithelial/endothelial cells

One of the most important possible consequences of lung infection is the destruction of the epithelial/endothelial cells forming the barrier between lung and circulation allowing for gas exchange. In the course of the disease, these cells are damaged, both by attacking pneumococci and by infiltrating immune cells as well. In consequence, the barrier becomes more permeable to cells and molecules. This can further promote systemic infection and inflammation up to sepsis. Similar to our former modeling [5] but in more detail, we incorporate these mechanisms into the model. The compartment EU consists of unaffected epithelial and endothelial cells, which are not distinguished. In the presence of bacteria, unaffected epithelial cells EU are transformed into affected epithelial cells EA with rate parameter *k*_EU_P_. Independent of bacteria, we further assume an additional barrier injury caused by neutrophil migration, suicidal netosis and other processes [7, 8] with rate parameter *k*_EU_N_. Thus

$$\begin{array}{ccc} \frac{dEU(t)}{dt} & = & -\left(k_{\text{EU}\_P}\cdot P\left(t\right)\cdot EU(t)+k_{\text{EU}\_N}\cdot EU(t)\cdot N\left(t\right)\right)+P_{\text{EU}}\\ P_{\text{EU}} & = & k_{\text{EU}\_N}\cdot EU_{0}\cdot N_{0} \end{array}$$ (6)

As a consequence of the above mentioned mechanisms, even in the disease free steady-state there is a constant loss of epithelial cells which is balanced by the constant production term *P*_EU_. This also ensures that the system returns to steady-state after elimination of pneumococci [9].

The compartment EA describes affected epithelial cells and is fed by cells from EU attacked by pneumococci or impaired by neutrophils [7, 8]. The influence of cytokines or other immune cells on EA is neglected. Cells in this compartment were removed with rate *d*_*E*_. Referring to data and the above mentioned mechanisms, we assume a steady state level *EA*_0_ > 0 caused by a constant “production” of such cells P_EA_.

$$\begin{array}{ccc} \frac{dEA(t)}{dt} & = & P_{\text{EA}}-d_{E}\cdot EA(t)+(k_{\text{EU}\_P}\cdot EU(t)+k_{\text{EU}\_N}\cdot EU(t)\cdot N\left(t\right)\\ P_{\text{EA}} & = & d_{E}\cdot {EA}_{0}-k_{\text{EU}\_N}\cdot EU_{0}\cdot N_{0} \end{array}$$ (7)

#### NOX-D19 treatment

The complement component C5a is involved in the immune response to infection, but it can also cause hyperinflammation or barrier and organ failure. NOX-D19 Spiegelmer was developed to neutralise C5a [2–4] which is intended to prevent or ameliorate these complications. We include this effect phenomenologically by modifying the flux from EU to EA with a NOX-D19-dose dependent sigmoidal function *ZD19*(*t*).

$$\begin{array}{l} ZD19(t)\\ =\text{ZD}19_{\text{max}}-(\text{ZD}19_{\text{max}}-\text{ZD}19_{\text{min}})e(-ln(\frac{\text{ZD}19_{\text{max}}-\text{ZD}19_{\text{min}}}{\text{ZD}19_{\text{max}}-\text{ZD}19_{\text{nor}}})\cdot (D19(t)){}^{\text{ZD}19_{\text{b}}}) \end{array}$$ (8)

*D19*(*t*) is set to the dose of NOX-D19 for 24 hours after application, and to zero otherwise. To include this effect, we extend Eqs 6 and 7, accordingly.

$$\begin{array}{ccc} \frac{dEU(t)}{dt} & = & -\left(k_{\text{EU}\_P}\cdot P\left(t\right)\cdot EU(t)+k_{\text{EU}\_N}\cdot EU(t)\cdot N\left(t\right)\right)\cdot ZD19(t)+P_{\text{EU}} \end{array}$$ (9)

$$\begin{array}{ccc} \frac{dEA(t)}{dt} & = & (k_{\text{EU}\_P}\cdot P\left(t\right)\cdot EU(t)+k_{\text{EU}\_N}\cdot EU(t)\cdot N\left(t\right))\cdot ZD19(t)\\ & & +P_{\text{EA}}-d_{E}\cdot EA(t) \end{array}$$ (10)

#### Compartment CFU-B

With increasing bacterial load and epithelial defects, bacteria or molecules can enter the bloodstream resulting in systemic infection [10–12]. In our model, transition of bacteria from alveolar spaces to blood depends on the number of affected epithelial cells and the amount of bacteria in alveolar spaces coupled by rate parameter *k*_CFUB_P_. Passage of bacteria through the barrier is modelled by two delay compartments:

$$\begin{array}{c} \frac{dC_{P\_\text{CFUB}}^{\left(i\right)}\left(t\right)}{dt}=C_{P\_\text{CFUB}\_\text{out}}^{\left(i-1\right)}\left(t\right)-k_{P\_\text{CFUB}}^{\text{Delay}}\cdot C_{P\_\text{CFUB}}^{\left(i\right)}\left(t\right)\;i=1,2 \end{array}$$ (11)

$$\begin{array}{ccc} C_{P\_\text{CFUB}\_\text{out}}^{\left(i\right)}\left(t\right) & = & k_{P\_\text{CFUB}}^{\text{Delay}}\cdot C_{P\_\text{CFUB}}^{\left(i\right)}\left(t\right)\;i=1,2\\ C_{P\_\text{CFUB}\_\text{out}}^{\left(0\right)}\left(t\right) & = & k_{\text{CFUB}\_P}\cdot P\left(t\right)\cdot EA\left(t\right) \end{array}$$

where $C_{P\_\text{CFUB}\_\text{out}}^{\left(0\right)}\left(t\right)$ is the efflux from compartment *P* and $C_{P\_\text{CFUB}\_\text{out}}^{\left(2\right)}\left(t\right)$ is the influx into the peripheral blood compartment *CFUB*:

$$\begin{array}{ccc} \frac{dCFUB(t)}{dt} & = & C_{P\_\text{CFUB}\_\text{out}}^{\left(2\right)}\left(t\right)-d_{\text{CFUB}}\cdot CFUB(t) \end{array}$$ (12)

Here, we only assume an unspecific removal of bacteria from blood stream with rate *d*_CFUB_, i.e. proliferation of bacteria, action of antibiotic treatment and immune responses are not explicitly modelled in the blood compartment.

#### Compartment N: Neutrophils

After alveloar macrophage, neutrophils represent the second line of the innate immune response to lung infection. Recruited by pro-inflammatory cytokines and chemokines, they migrate into the alveolar space to eliminate bacteria [7]. As in our former model [5], we assume a positive correlation of neutrophil migration with the cytokine IL-6 saturated by a maximum number *N*_max_ [6, 13]. *N*_max_ is set to the highest neutrophil value observed in the experimental group without treatment. Here, we additionally assume a positive correlation with the chemokines CXCL1 and CXCL5 [14]. Neutrophils are lost due to fighting the infection at rate *d*_N_P_ or by natural cell death at rate *dN* [6]. A neutrophil count of *N*_0_, realized by a baseline recruitment P_*N*_, is assumed as disease-free steady state.

$$\begin{array}{lll} \frac{dN(t)}{dt} & = & \left(k_{\text{N}\_\text{IL6}}\cdot IL6(t)+k_{\text{N}\_\text{CXCL5}}\cdot CXCL5(t\right)\\ & & +k_{\text{N}\_\text{CXCL1}}\cdot CXCL1(t))\cdot (1-\frac{N(t)}{N_{\text{max}}})\\ & & -d_{\text{N}\_\text{P}}\cdot N(t)\cdot P(t)d_{N}\cdot N(t)+{\text{P}}_{\text{N}}\\ {\text{P}}_{\text{N}} & = & d_{N}\cdot N_{0}-(k_{\text{N}\_\text{IL6}}\cdot IL6_{0}+k_{\text{N}\_\text{CXCL5}}\cdot CXCL5_{0}\\ & & +k_{\text{N}\_\text{CXCL1}}\cdot CXCL1_{0})\cdot (1-\frac{N_{0}}{N_{\text{max}}}) \end{array}$$ (13)

#### Compartment MONO: Blood monocytes

As third line of immune response, monocytes migrate to the alveolar space and differentiate into inflammatory macrophages. These processes are promoted by IL-6 [15] and CCL2 [16]. The antiinflammatory cytokine IL-10 counter-acts monocyte recruitment which is modelled by a dose-dependent inverse Z-function *ZIL*10 (see below).

$$\begin{array}{ccc} & & \frac{d\text{MONO}(t)}{dt}=k_{\text{MONO}\_\text{IL}6}\cdot \text{IL}6\left(t\right)\cdot \text{ZIL}10\left(t\right)\\ & & -(k_{\text{MONO}\_\text{IM}\_\text{IL}6}\cdot \text{IL}6\left(t\right)+k_{\text{MONO}\_\text{IM}\_\text{CCL}2}\cdot CCL2\left(t\right))\cdot \text{MONO}\left(t\right)\\ & & -d_{\text{MONO}}\cdot \text{MONO}\left(t\right)+P_{\text{MONO}} \end{array}$$ (14)

with steady state condition

$$\begin{array}{ccc} P_{\text{MONO}} & = & k_{\text{MONO}\_\text{IM}\_\text{IL}6}\cdot {\text{MONO}}_{0}\cdot \text{IL}6_{0}+d_{\text{MONO}}\cdot {\text{MONO}}_{0}\\ & & -k_{\text{MONO}\_\text{IL}6}\cdot \text{IL}6_{0}\cdot \text{ZIL}10_{0} \end{array}$$

We assume that monocyte to macrophage differentiation occurs with some time delay modeled by a delay compartment *C*_M_(*t*):

$$\begin{array}{c} \frac{dC_{M}\left(t\right)}{dt}=C_{\text{MONO}\_\text{out}}\left(t\right)-k_{\text{MONO}}^{\text{Delay}}\cdot C_{M}\left(t\right) \end{array}$$ (15)

with the influx

$$\begin{array}{c} C_{\text{MONO}\_\text{out}}\left(t\right)=\left(k_{\text{MONO}\_\text{IM}\_\text{IL}6}\cdot \text{IL}6\left(t\right)+k_{\text{MONO}\_\text{IM}\_\text{CCL}2}\cdot CCL2\left(t\right)\right)\cdot \text{MONO}\left(t\right) \end{array}$$

and the efflux

$$\begin{array}{c} {\text{MONO}}_{\text{out}}\left(t\right)=k_{\text{MONO}}^{\text{Delay}}\cdot C_{M}\left(t\right) \end{array}$$

entering the compartment of inflammatory macrophages IM.

#### Compartments of alveolar macrophages (AM) and inflammatory macrophages (IM)

Alveolar macrophages AM serve as first line of innate immune response [17]. In contrast to our earlier model, an improved data base now allows us to distinguish between alveolar macrophages AM and migrated monocyte derived macrophages IM. Analogeous to Smith et al. [6], losses of macrophages due to fighting the infection are neglected. Accordingly, the number of alveolar macrophages is assumed to be constant. However, for the inflammatory macrophages, we assume a reduction rate *d*_IM_ to allow re-establishment of the disease-free steady state after successful elimination of the infection.

$$\begin{array}{c} \frac{d\text{IM}(t)}{dt}=k_{\text{MONO}}\cdot {\text{MONO}}_{\text{out}}\left(t\right)-d_{\text{IM}}\cdot \text{IM}\left(t\right)+P_{\text{IM}} \end{array}$$ (16)

with the steady state condition

$$\begin{array}{c} P_{\text{IM}}=d_{\text{IM}}\cdot {\text{IM}}_{0}-k_{\text{MONO}}\cdot k_{\text{MONO}\_\text{IM}\_\text{IL}6}\cdot IL6_{0}\cdot {\text{MONO}}_{0}. \end{array}$$

#### Compartment IL6: Cytokine IL-6

The cytokine IL-6 is produced by several cells, including affected epithelial cells and macrophages [18, 19]. IL-6 is known for its various proinflammatory effects, for instance its involvement in differentiation, maturation and activation of immune cells [19]. IL-6 serves as the most important mediator of immune response in our model which is based on the following assumptions. IL-6 attracts cells of the immune system (monocytes, neutrophils) and stimulates the differentiation of monocytes into macrophages. It is secreted by affected epithelial cells at rate *k*_IL6_EA_ and by alveloar and inflammatory macrophages at rates *k*_IL6_AM_ and *k*_IL6_IM_, resepectively. IL-6 secretion of affected epithelial cells starts immediately, whereas we assume a delayed production of IL-6 by macrophages resulting in slower increase in case of infection and a later stop of production in case of removal of the infection. This is modeled by inserting a delay compartment.

$$\begin{array}{ccc} \frac{dC_{\text{delay}\_\text{IL}6}\left(t\right)}{dt} & = & \left(k_{\text{IL}6\_\text{AM}}\cdot {AM}_{0}+k_{\text{IL}6\_\text{IM}}\cdot \text{IM}\left(t\right)\right)\cdot P\left(t\right)-k_{P}^{\text{Delay}}\cdot C_{\text{delay}\_\text{IL}6}\left(t\right)\\ C_{\text{delay}\_\text{IL}6}\left(0\right) & = & 0 \end{array}$$

The efflux $k_{P}^{\text{Delay}}\cdot C_{\text{delay}\_\text{IL}6}\left(t\right)$ of this compartment enters the compartment IL6. We further assume a constant baseline production P_IL6_ and a first order elimination at rate *d*_IL6_.

$$\begin{array}{ccc} \frac{\text{dIL}6(t)}{dt} & = & k_{\text{IL}6\_\text{EA}}\cdot EA\left(t\right)+k_{P}^{\text{Delay}}\cdot C_{\text{delay}\_\text{IL}6}\left(t\right)-d_{\text{IL}6}\cdot \text{IL}6\left(t\right)+P_{\text{IL}6}\\ P_{\text{IL}6} & = & d_{\text{IL}6}\cdot \text{IL}6_{0}-k_{\text{IL}6\_\text{EA}}\cdot {EA}_{0} \end{array}$$ (17)

#### Compartment IL10: Cytokine IL-10

The anti-inflammatory cytokine IL-10 reduces the activity of several immune cells and the production of different proinflammatory cytokines. It is a major down-regulator of the immune response required to stop immune reaction after successfully fighting the infection. Most cells of the immune system are able to produce IL-10 [19, 20]. For our modeling, it is sufficient to assume production by monocytes in peripheral blood with rate *k*_IL10_MONO_ and by neutrophils in alveolar spaces with rate *k*_IL10_N_. An unspecific elimination with rate *d*_IL10_ and a constant baseline production P_IL10_ implies a positive level of IL10 at disease-free steady-state.

$$\begin{array}{ccc} \frac{d\text{IL}10(t)}{dt} & = & k_{\text{IL}10\_\text{MONO}}\cdot \text{MONO}\left(t\right)+k_{\text{IL}10\_N}\cdot N\left(t\right)-d_{\text{IL}10}\cdot \text{IL}10\left(t\right)+P_{\text{IL}10}\\ P_{\text{IL}10} & = & d_{\text{IL}10}\cdot {\text{IL}10}_{0}-k_{\text{IL}10\_N}\cdot N_{0}-k_{\text{IL}10\_\text{MONO}}\cdot {\text{MONO}}_{0} \end{array}$$ (18)

The anti-inflammatory effect of IL-10 on monocytes and CCL2 is modeled by a sigmoidal function multiplied to several terms representing immune response.

$$\begin{array}{ccc} & & Z\_\text{IL}10={Z\_\text{IL}10}_{\text{max}}\\ & & -\left({Z\_\text{IL}10}_{\text{max}}-{Z\_\text{IL}10}_{\text{min}}\right)\cdot \exp(-\ln(\frac{{Z\_\text{IL}10}_{\text{max}}-{Z\_\text{IL}10}_{\text{min}}}{{Z\_\text{IL}10}_{\text{max}}-{Z\_\text{IL}10}_{\text{nor}}})\cdot {\left({\frac{\text{IL}10}{\text{IL}10}}_{0}\right)}^{Z\_\text{IL}10_{b}}) \end{array}$$ (19)

where different sensitivities Z_IL10_*b*_ are assumed for monocytes, respectively CCL2.

#### Compartment TNFa: Tumour necrosis factor TNF-*α*

The tumour necrosis factor TNF-*α* is another major cytokine of immune response with various pro-inflammatory effects. It is secreted mainly by macrophages [19]. Therefore, we decided to include TNF-*α* into our model as a potent marker of the third line of immune response and to compare it with available data. To limit the complexity of the model, the multiple effects of TNF-*α* on other components of the immune system are neglected, i.e. TNFa serves as an outcome parameter of our model with no feedback to other state variables. Again, a delayed production is assumed. Parameters are set to those of IL-6 for model parsimony.

$$\begin{array}{ccc} \frac{dC_{\text{delayTNFa}}\left(t\right)}{dt} & = & \left(k_{\text{TNFa}\_\text{AM}}\cdot {AM}_{0}+k_{\text{TNFa}\_\text{IM}}\cdot \text{IM}\left(t\right)\right)\cdot P\left(t\right)-k_{P}^{\text{Delay}}\cdot C_{\text{delayTNFa}}\left(t\right)\\ C_{\text{delayTNFa}}\left(0\right) & = & 0 \end{array}$$ (20)

The efflux *k*_delayP_ ⋅ *C*_delayTNFa_(*t*) of this compartment enters the compartment TNFa.

$$\begin{array}{ccc} \frac{\text{dTNFa}(t)}{dt} & = & k_{\text{TNFa}\_\text{EA}}\cdot EA\left(t\right)+k_{\text{delayP}}\cdot C_{\text{delayTNFa}}\left(t\right)-d_{\text{TNFa}}\cdot TNFa\left(t\right)+P_{\text{TNFa}}\\ P_{\text{TNFa}} & = & d_{\text{TNFa}}\cdot {\text{TNFa}}_{0}-k_{\text{TNFa}\_\text{EA}}\cdot {EA}_{0} \end{array}$$ (21)

#### Compartment CCL2: Monocyte chemoattractant protein-1 (MCP-1)

Monocyte chemoattractant protein-1 (MCP-1,CCL2) stimulates the migration of monocytes to the site of inflammation [21]. We assume CCL2 to be produced by the recruited monocytes [16], triggered by affected epithelial cells at rate *k*_CCL2_MONO_. The contribution of other cell types to CCL2 production is neglected. IL-10 suppresses the production of CCL2 as modeled by the sigmoidal function *ZIL10*(*t*) (see above).

$$\begin{array}{ccc} \frac{dCCL2(t)}{dt} & = & k_{\text{CCL}2\_\text{MONO}}\cdot \text{MONO}\left(t\right)\cdot (\frac{EA(t)}{{EA}_{0}}-1)\cdot \text{ZIL}10(t)-d_{\text{CCL}2}\cdot CCL2\left(t\right) \end{array}$$ (22)

with *CCL2*(0) = 0.

#### Compartment CXCL1: Chemokine (C-X-C motif) ligand 1 (GROa)

The chemokine CXCL1 (GROa) attracts neutrophils and is of high importance in the murine immune response [22]. It is produced by affected epithelial cells, neutrophils and macrophages [14, 23]. The production by macrophages is assumed to be delayed in analogy to IL-6 or IL-10 production:

$$\begin{array}{ccc} \frac{dC_{\text{delayCXCL}1}\left(t\right)}{dt} & = & (k_{\text{CXCL}1\_\text{AM}}\cdot {AM}_{0}+k_{\text{CXCL}1\_\text{IM}}\cdot \text{IM}\left(t\right))\cdot P\left(t\right)\\ & & -k_{P}^{\text{Delay}}\cdot C_{\text{delayCXCL}1}\left(t\right)\\ C_{\text{delayCXCL}1}\left(0\right) & = & 0 \end{array}$$ (23)

This results in the following balance equation for CXCL1.

$$\begin{array}{ccc} \frac{\text{dCXCL}1(t)}{dt} & = & k_{\text{CXCL}1\_\text{EA}}\cdot EA\left(t\right)+k_{\text{CXCL}1\_N}\cdot N\left(t\right)\\ & & +k_{P}^{\text{Delay}}\cdot C_{\text{delayCXCL}1}\left(t\right)-d_{\text{CXCL}1}\cdot CXCL1\left(t\right)+P_{\text{CXCL}1}\\ P_{\text{CXCL}1} & = & d_{\text{CXCL}1}\cdot \text{CXCL}1_{0}-k_{\text{CXCL}1\_\text{EA}}\cdot {EA}_{0}-k_{\text{CXCL}1\_N}\cdot N_{0} \end{array}$$ (24)

#### Compartment CXCL5: Chemokine (C-X-C motif) ligand 5 (LIX, ENA78)

The chemokine CXCL5 (LIX, ENA78) attracts neutrophils and it is one of the most dominant chemokines in the mouse [14]. CXCL5 is produced exclusively by affected epithelial cells. Thus, it represents epithelial activation [14, 24, 25].

$$\begin{array}{ccc} \frac{\text{dCXCL}5(t)}{dt} & = & k_{\text{CXCL}5\_\text{EA}}\cdot EA\left(t\right)-d_{\text{CXCL}5}\cdot \text{CXCL}5\left(t\right)+P_{\text{CXCL}5}\\ P_{\text{CXCL}5} & = & d_{\text{CXCL}5}\cdot \text{CXCL}5_{0}-k_{\text{CXCL}5\_\text{EA}}\cdot {EA}_{0} \end{array}$$ (25)

#### Compartment D: Debris

Apoptotic neutrophils, eliminated bacteria and affected epithelial cells are summarized as debris [6] contributing with rates *k*_D_N_, *k*_D_N_P_ and *k*_D_EA_, respectively. Debris is reduced with constant rate *d*_*D*_. In our data, debris is measured as histological semi-quantitative score evaluating purulent and catarrhalic pneumonia, perivascular edema, alveolar edema, and inflammation of pleura and mediastinal adipose tissue [26].

$$\begin{array}{c} \frac{\text{dD}(t)}{dt}=\;k_{D\_N\_P}\cdot N\left(t\right)\cdot P\left(t\right)+k_{D\_N}\cdot N\left(t\right)+k_{D\_\text{EA}}\cdot EA\left(t\right)-d_{D}\cdot D\left(t\right) \end{array}$$ (26)

with

$$\begin{array}{c} d_{D}=\frac{k_{D\_N}\cdot N_{0}+k_{D\_\text{EA}}\cdot EA_{0}}{D_{0}} \end{array}$$

In our model, debris serves as an outcome parameter not affecting other compartments.

### Numerical methods for simulation

Differential equations are implemented in MATLAB 7.5.0.342 (R2007b) using the SIMULINK toolbox (The MathWorks Inc., Natick, MA, USA). Numerical solutions of the equation system are obtained using the variable step solver from Adams and Bashford (ode113, SIMULINK toolbox).

### Data

Model simulations are compared with comprehensive data from mice experiments comprising time series of pneumococci, measured as colony-forming units in blood and BAL, neutrophils, alveolar and inflammatory macrophages in BAL, IL-6, TNF-*α*, IL-10, CCL2 (MCP-1), CXCL5 (LIX, ENA78), and CXCL1 (GROa) measured in bronchoalveolar lavage fluid (BALF) and barrier function (measured by the ratio of murine serum albumin (MSA) in BALF and serum/plasma). In the antibiotics experiments, data of time points without antibiotic treatment were pooled across experimental groups.

#### Experiments

All animal studies have been ethically reviewed and approved by the animal ethics committee of Charité—Universitätsmedizin Berlin and local governmental authorities (Landesamt für Gesundheit und Soziales Berlin).

Mice were transnasally inoculated with 5 ⋅ 10^6^ colony-forming units (CFUs) of *Streptococcus pneumoniae* (serotype 3, strain PN36, NCTC 7978). In the antibiotics experiments, starting at 24 h or 48 h p.i., ampicillin (0.4 mg/mouse corresponding to 0.02mg/g body weight) was injected intraperitoneally every 12 h (see [27]. A detailed description of the pneumococcal pneumonia model and corresponding data of antibiotic experiments are published in [26].

For C5a neutralization, mice were injected intraperitoneally with anti-C5a L-RNA-aptamer NOX-D19 (20mg/kg or 2mg/kg) or solvent (5% glucose) at the time of infection (0h) and 24 h post infection. This dose was chosen on the basis of pharmacokinetic data of NOX-D20, which is similar to NOX-D19 [4]. The dose of 2mg/kg showed no reduction in pulmonary permeability. Therefore, the dose of 20mg/kg was considered primarily. Treatment with NOX-D19 did not affect leukocyte counts in alveolar spaces and blood, cytokine levels in BALF and blood besides lower G-CSF levels in blood 48h post infection. NOX-D19 had no impact on the bacterial load in alveolar spaces, spleen, liver and blood. A detailed description of these data has been published separately [3].

Mice were euthanized 24h or 48h post infection. Sham infected mice were euthanized 24h post sham infection. Pulmonary permeability was assessed by diffusion of intravenously injected human serum albumin into the alveolar space as described previously [28]. Neutrophil, macrophage, and lymphocyte counts were determined in BAL, neutrophil, monocyte and lymphocyte counts were determined in blood. Cytokine levels in BALF and blood were analyzed by multiplex ELISA technique as described previously [27].

#### Comparison of model compartments and measured quantities

To compare model and data, we used appropriate counter parts of measured features and simulated model compartments (see Table 1).

**Table 1:** Data and corresponding model compartments.

model compartment	experimental read-out
P (pneumococci)	pneumococci in BAL
CFUB (pneumococci)	pneumococci in blood
IL-6 (cytokine)	IL-6 measured in BALF
IL-10 (cytokine)	IL-10 measured in BALF
TNFa	TNF-*α* measured in BALF
CCL2	CCL2 measured in BALF
CXCL1	CXCL1 measured in BALF
CXCL5	CXCL5 measured in BALF
N (neutrophils)	neutrophils in BAL
AM (macrophages)	alveolar macrophages in BAL
IM (macrophages)	inflammatory macrophages in BAL
MONO (monocytes)	monocytes in blood
EA (affected epithelial cells)	proxied by ratio BALF/serum albumine

Steady state values are derived from the data by calculating geometric means of initial values over all experimental groups (see Table in S1 File).

### Estimation of parameters

Model calibration is performed by optimizing the agreement of simulation results and data applying the following goal function.

$$\begin{array}{c} \sum_{j=1}^{L}{\left(\frac{f_{\text{model}}\left(t_{j},k\right)-f_{\text{data}}\left(t_{j}\right)}{\sigma_{j}}\right)}^{2}\to \min_{k}, \end{array}$$ (27)

Here, *f*_*model*_(*t*, **k**) represents the solution of the differential equation system with the parameter set **k** = *k*_1_, …*k*_*n*_ at time points *t*_*j*_ supported by data, and *f*_*data*_(*t*_*j*_) are means of measured values at time *t*_*j*_, *j* = 1, …, *L*, with corresponding standard deviation *σ*_*j*_. The left hand side of Eq (27) is referred as the fitness function in the following. Simultaneous fitting of different data sets is achieved by adding respective fitness functions.

To solve the optimization problem, we relied on (1+3)-evolutionary-strategies with self-adapting mutation step size (see [29, 30]) as in our previous work [5]. We initially fitted single differential equations to reduce the number of simultaneous parameter fittings. This is achieved by imposing data curves of coupled state variables rather than model curves. In the next step, we coupled all equations and refined the parameter estimation.

A list of the estimated parameters can be found in S2-S4 Tables in S1 File.

## Results

### General model behavior

**Fig 3:**
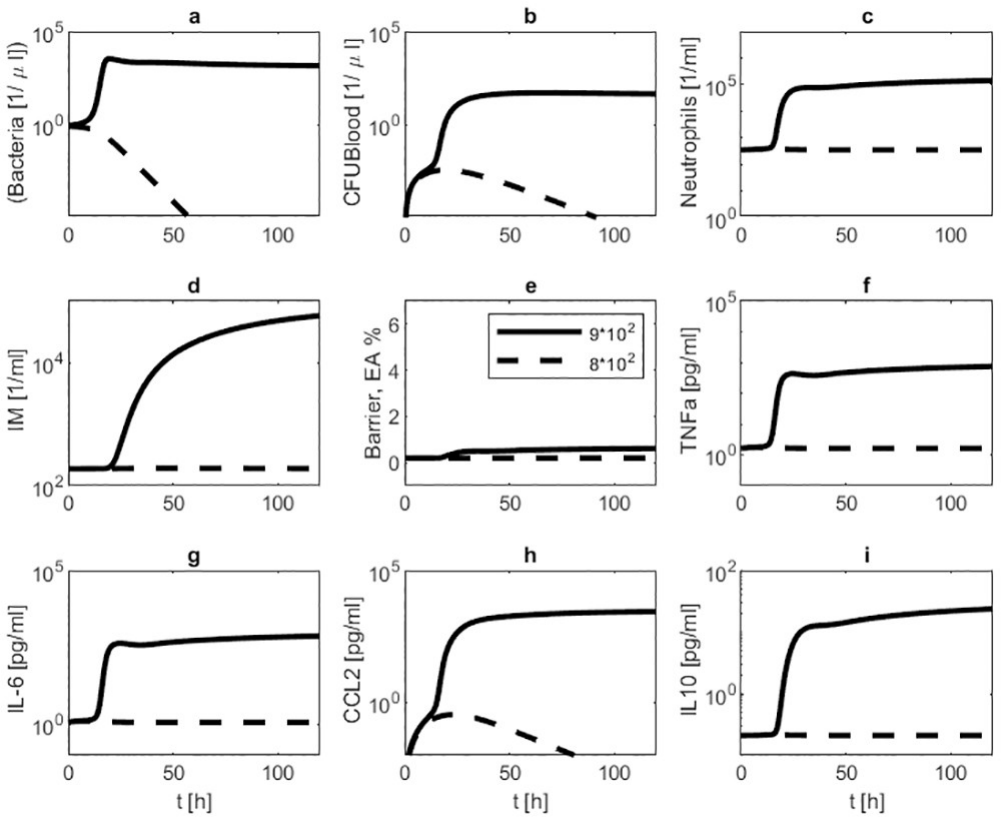
Model behaviour in dependence on initial bacterial load. We present simulation results of infections with 8 ⋅ 10^2^ (dotted line) respectively 9 ⋅ 10^2^ (solid line) seeded *Streptococcus pneumoniae* without treatment. Time series of pneumococcal population in BAL (a) and blood (b), neutrophils in BAL (c), inflammatory macrophages in BAL (d), barrier function (e), TNF-*α* in BALF (f), IL-6 in BALF (g), CCL2 in BALF (h) and IL-10 in BALF (i) are displayed. The lower infectious dose results in cure while the larger infectious dose leads to disseminated disease.

We first performed simulations of different initial bacterial loads without treatments to study the general model behaviour. In case of low initial load, bacteria are successfully eliminated by the immune system, and the model returns to it’s steady state. Higher doses of bacteria result in more severe infections, and eventually, the system fails to eliminate the infection. Results are displayed in Fig 3.

### Parameter sensitivity

Our model contains 66 parameters without therapy and 9 intervention-related parameters. 50 of these parameters (45 of the baseline model, five intervention-related) were identified by fitting the predictions of the model to available data, while the other parameters are set either to known values or to plausible values in case of low identifiability. Fitted parameters were subjected to a comprehensive sensitivity analysis. For this purpose, we evaluated deteriorations of the fitness function when changing single parameter values by ±10% keeping the other parameters constant (see S1 Fig in S1 File).

The most sensitive parameters are the initial growth rate of bacteria (*k*_p_), the degradation rate of CCL2 (*d*_CCL2_), the contribution to debris from epithelial cells and neutrophils (*k*_DEA_, *k*_DN_), the migration rate of bacteria through the epithelial barrier (*k*_CFUB_P_), the removal of macrophages (*d*_IM_), and the neutrophil recruitment rate by IL-6 (*k*_N_IL6_).

The sensitivity of monocyte recruitment suppression by IL-10 (*ZIL10_MONO*_b_) and the production rates of CXCL1 by macrophages and neutrophils (*k*_CXCL1_IM_, *k*_CXCL1_N_) are the parameters with lowest sensitivity.

Similar results were obtained when considering other measures of parameter sensitivity. We analysed the impact of relative changes of model parameters on the fitness function. This reveals, for example, that for certain parameters (e.g(e.g. *k_DN_*, *d*_*IL*10_) only upper or lower bounds are well identifiable (see S2 Fig in S1 File). We also simulated the certainty of model predictions if parameters are sampled with a 10% variance (see S3, S4 Figs in S1 File).

### Comparison of model and data

**Fig 4:**
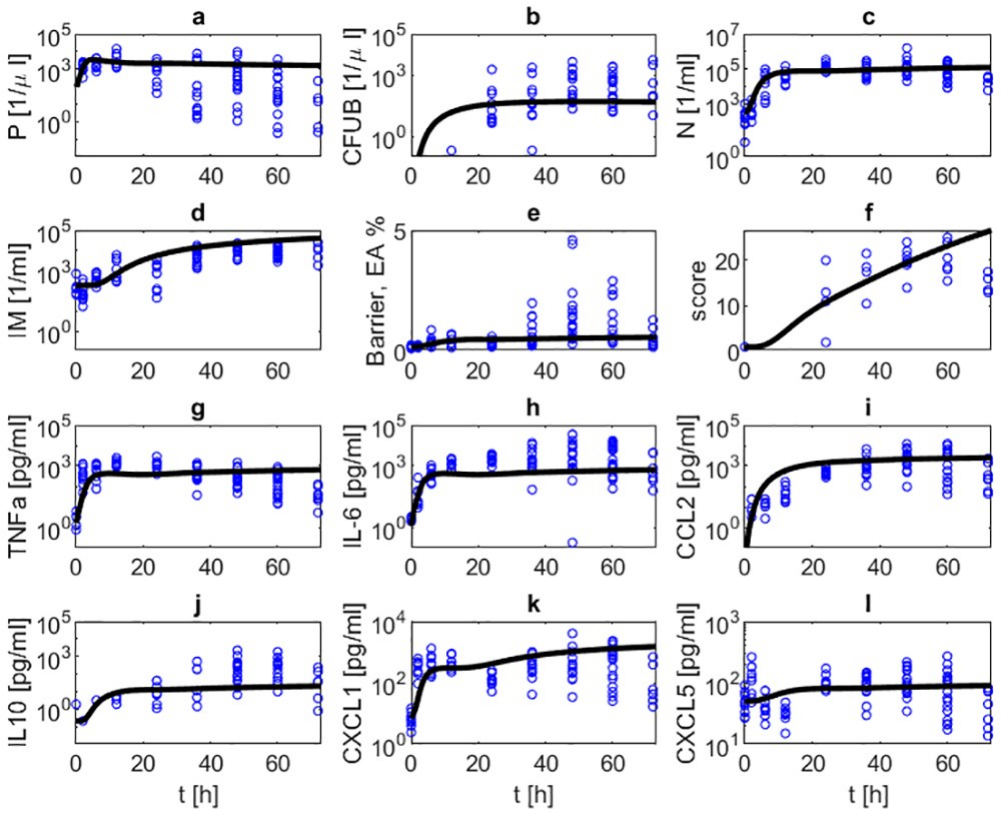
Comparison of model and data for a scenario of infection with 5 ⋅ 10^6^. ***Streptococcus pneumoniae*** **without treatment.** Time series of pneumococcal population in BAL (a) and blood (b), neutrophils in BAL (c), inflammatory macrophages in BAL (d), barrier function (e), histologic score (f), TNF-*α* in BALF (g), IL-6 in BALF (h), CCL2 in BALF (i), IL-10 in BALF (j), CXCL1 in BALF (k) and CXCL5 in BALF (l) without antibiotic treatment are displayed. The solid black curve represents simulation results. Circles represent data points. In this scenario, the immune system is not able to remove the infection resulting in increasing numbers of immune cells, pro-inflammatory cytokines, barrier failure and bacteremia.

Parameter estimates resulted in a good agreement of model and data for the majority of time points. In Fig 4, the simulated time courses of pneumococcal population in BAL and blood, neutrophils and inflammatory macrophages in BAL, TNF-*α*, IL-6, CCL2, IL-10, CXCL1 and CXCL5 in BALF, barrier function, and the histologic score after infection with 5 ⋅ 10^6^ *Streptococcus pneumoniae* without treatment are presented and compared with corresponding data. In this experimental scenario, the murine immune system is not able to successfully fight the infection and most animals had to be euthanized after 48h. Accordingly, data and corresponding simulations show that bacteria, cytokine levels and immune cell levels continuously rise or remain on high levels over the observation period of 48 hours. The barrier function (EA) deteriorates quickly after onset of infection and remains impaired over the observation period. Correspondingly, bacteremia shows a similar dynamics.

**Fig 5:**
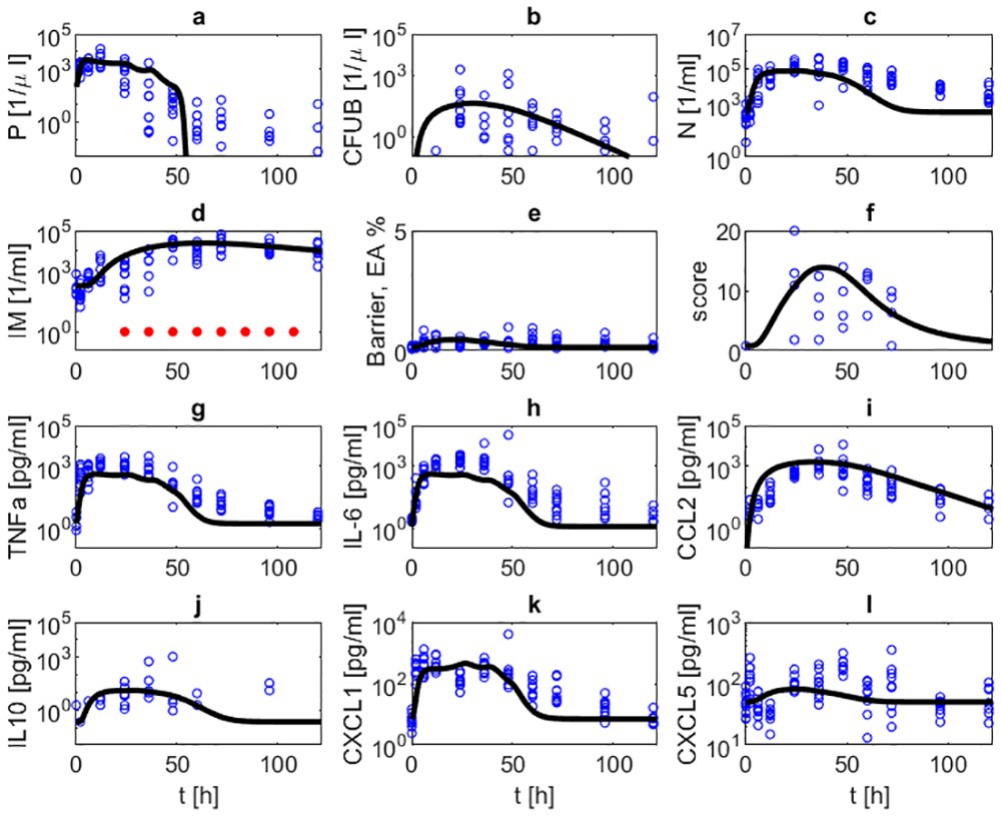
Infection with 5 ⋅ 10^6^ *Streptococcus pneumoniae* treated with ampicillin (0.4 mg/mouse) every 12h, starting at 24h. We compare results of model simulation with data under antibiotic treatment. Time series of pneumococcal population in BAL (a) and blood (b), neutrophils in BAL (c), inflammatory macrophages in BAL (d), barrier function (e), histologic score (f), TNF-*α* in BALF (g), IL-6 in BALF (h), CCL2 in BALF (i), IL-10 in BALF (j), CXCL1 in BALF (k) and CXCL5 in BALF (l) are displayed. The solid black curve represents simulation results. Blue circles represent data. Red dots represent the time points of antibiotic treatment. Treatment results in eradication of bacteria from alveolar spaces and blood. Immune cells, cytokine levels and barrier function also normalize except for macrophages staying elevated during observation period.

**Fig 6:**
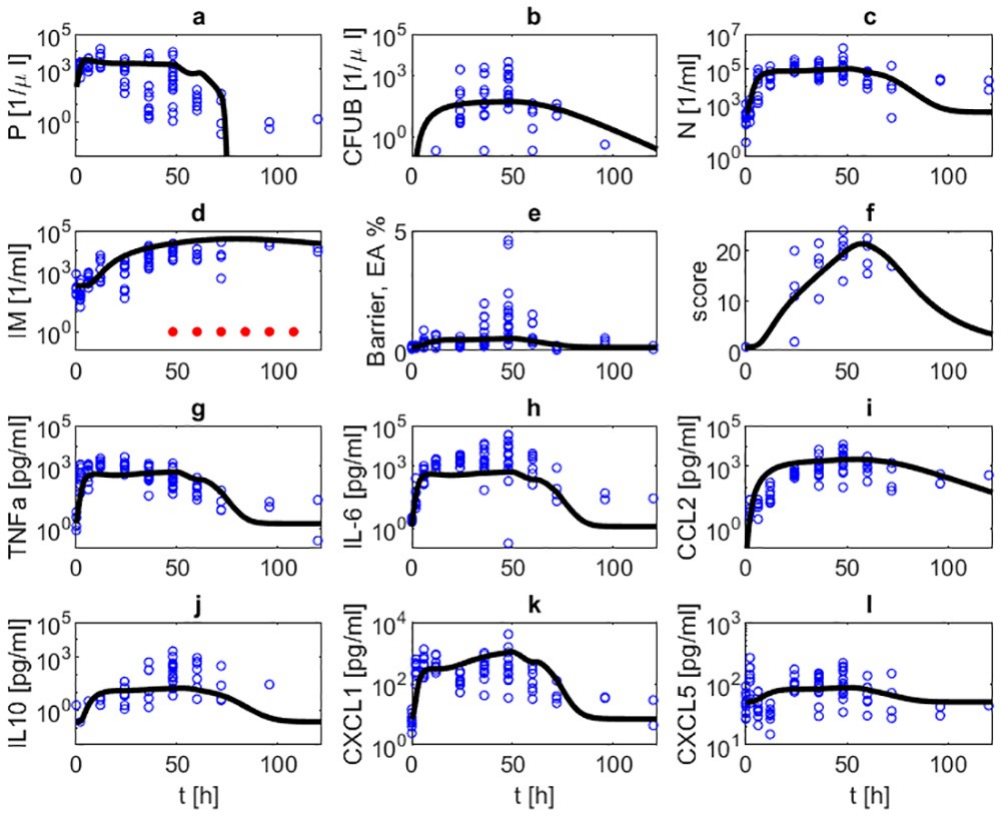
Infection with 5 ⋅ 10^6^ *Streptococcus pneumoniae* treated with ampicillin (0.4 mg/mouse) every 12h, starting at 48h. We compare results of model simulation with data under antibiotic treatment. Time series of pneumococcal population in BAL (a) and blood (b), neutrophils in BAL (c), inflammatory macrophages in BAL (d), barrier function (e), histologic score (f), TNF-*α* in BALF (g), IL-6 in BALF (h), CCL2 in BALF (i), IL-10 in BALF (j), CXCL1 in BALF (k) and CXCL5 in BALF (l) are displayed. The solid black curve represents simulation results. Blue circles represent data. Red dots represent the time points of antibiotic treatment. Dynamics of state parameters are similar to that of antibiotic treatment starting at 24h but with some delay.

Next, we studied scenarios with antibiotic treatment. Figs 5 and 6 show corresponding comparisons of model simulations and data after infections with 5 ⋅ 10^6^ bacteria under treatment with ampicillin (0.4 mg/mouse) every 12 hours, either starting 24 hours or 48 hours after infection. In both scenarios, bacteria are eradicated and immune cell numbers and cytokine levels normalize. Numbers of bacteria in lung and blood are quickly reduced within three, respectively four days. Cytokine levels normalize within three to seven days, where IL-10 and CCL2 react with the largest delay. Neutrophil counts in lung are predicted to reach steady-state values after about four or five days. Normalization of macrophage counts is achieved much later. These predictions could be experimentally validated on the basis of longer time series.

**Fig 7:**
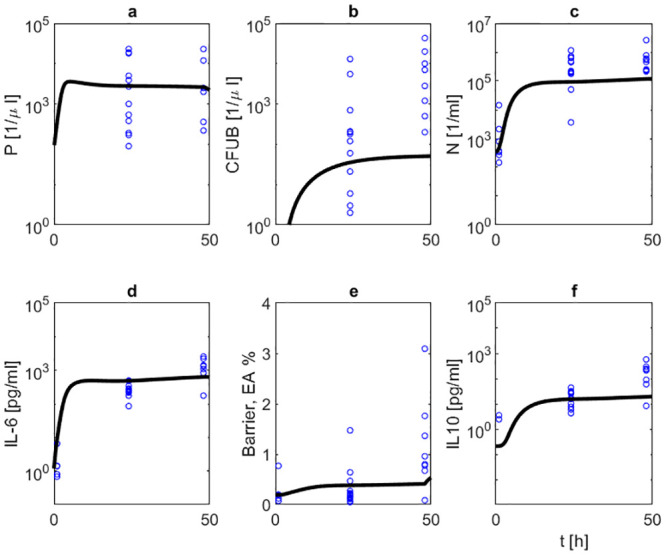
(Treatment with 2 mg/kg NOX-D19). We compare results of model simulation (solid black curve) with data under 2 mg/kg NOX-D19 treatment. Time series of pneumococcal population in BAL (a) and blood (b), neutrophils in BAL (c), IL-6 in BALF (d), barrier function (e) and IL-10 in BALF (f) are displayed. Circles represent data points [2]. NOX-D19 is applied 0 and 24 hours after infection. NOX-D19 treatment alone does not help to eradicate the disease but stabilizes the barrier function.

**Fig 8:**
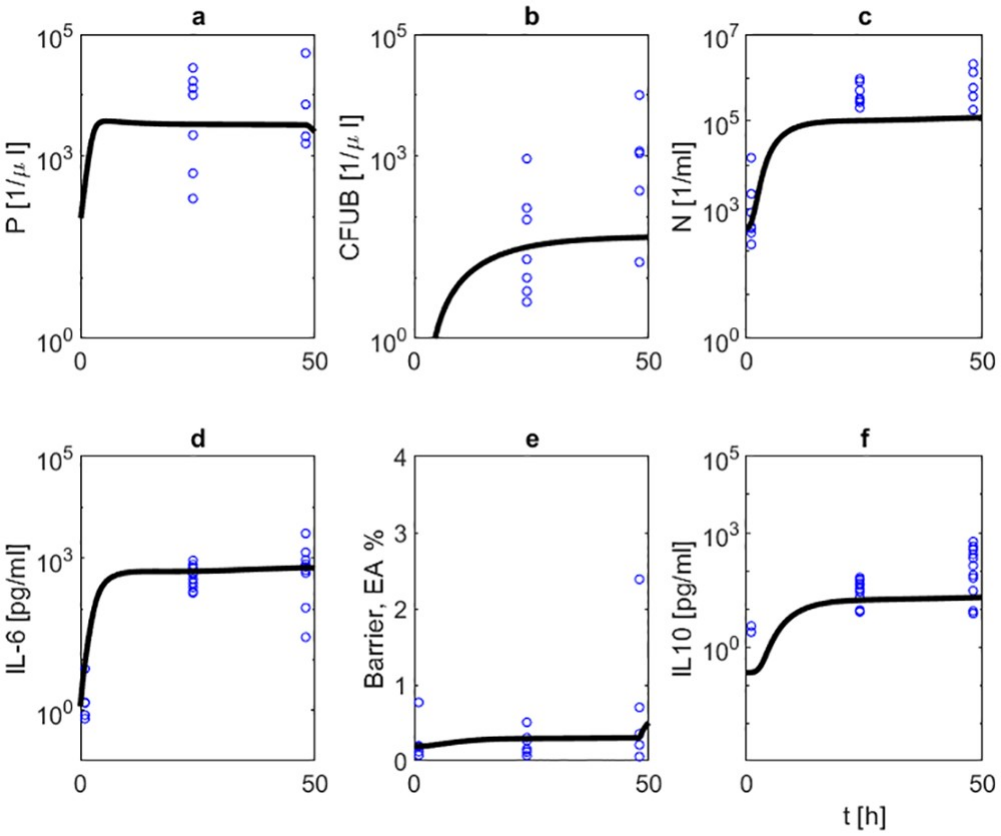
(Treatment with 20 mg/kg NOX-D19). We compare results of model simulation (solid black curve) with data under 20 mg/kg NOX-D19 treatment. Time series of pneumococcal population in BAL (a) and blood (b), neutrophils in BAL (c), IL-6 in BALF (d), barrier function (e) and IL-10 in BALF (f) are displayed. Circles represent data points [2]. NOX-D19 is applied 0 and 24 hours after infection. NOX-D19 alone is insufficient to eradicate the disease but stabilizes the barrier function.

We then studied the effects of NOX-D19 treatment. Two dose levels (low dose: 2 mg/kg, high dose: 20 mg/kg) were considered. NOX-D19 was applied directly at the time of infection and 24 hours later [2]. Figs 7 and 8 show comparisons of model simulations and data for these scenarios. NOX-D19 alone is insufficient to eliminate the disease but stabilizes the barrier function compared to no treatment.

### Model prediction

**Fig 9:**
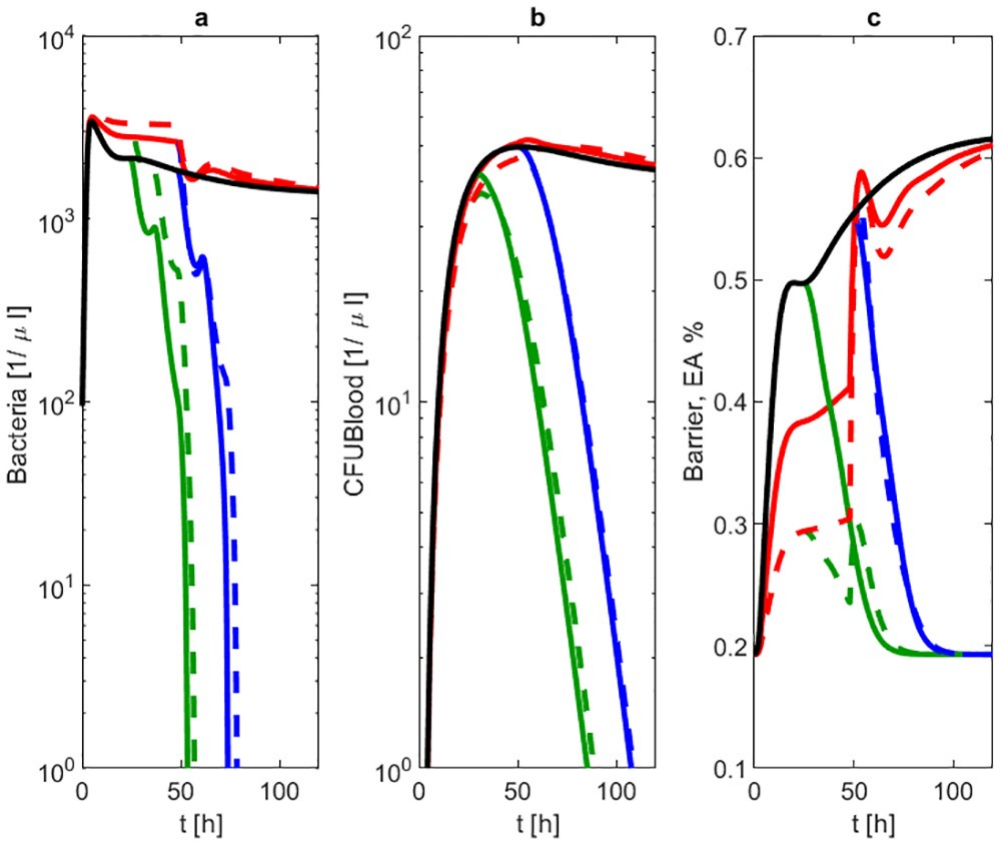
Prediction scenarios. We simulated the outcome of combined 20 mg/kg NOX-D19 and antibiotic treatment starting 24h (green dashed line) or 48h (blue dashed line) after infection and compared the results with no treatment (black solid line), antibiotic treatment alone 24h (green solid line) or 48h (blue solid line) after infection or 2 mg/kg (red solid line) respectively 20 mg/kg (red dashed line) NOX-D19 alone. Time series of pneumococcal population in BAL (a) and blood (b) and barrier function (c) are displayed. Cure is only achieved in scenarios with antibiotic treatment. Bacteremia is smallest in case of 20 mg/kg NOX-D19 combined with early antibiotic treatment.

After calibrating the model, we now demonstrate how it can be used for prediction purposes. Since no data of combined antibiotic and NOX-D19 therapy are available, we simulate such scenarios for later validation by experimental sudies. In detail, we combine the antibiotic treatments studied above with the 20 mg/kg NOX-D19 treatment given 0 and 24 hours after infection. The antibiotic therapy eradicates pneumococci in the lung. Additional application of 20 mg/kg D19 is predicted to result in approximately 45% improvement of barrier function and 10% reduction in bacteremia as determined on the basis of the respective extreme values within the first 48h (see S5 Table in S1 File). After stopping NOX-D19 therapy, barrier destruction continues. Overall, best outcome is achieved by combining 20 mg/kg NOX-D19 with antibiotic treatment started at 24 hours (see Fig 9).

## Discussion

Understanding disease dynamics and underlying patho-mechanisms is of utmost importance to improve outcome of pneumonia therapy. In addition to antibiotic therapy, novel adjunctive treatments are under development such as anti-C5a, trimodulin and vasculotide among others [3, 31, 32]. However, so far the most promising therapy schedule for a specific clinical situation is not known, for example how to combine antibiotics with adjunctive treatments. To support this developmental process, we here propose a biomathematical model of mechanisms of lung infection, immune responses, barrier function and therapy actions. The model allows predictions of therapy outcome for yet untested therapy options which could be used to select optimized schedules to be tested in follow-up experiments.

Based on improved experimental data and experiments with the novel anti-C5a drug NOX-D19, we comprehensively revised and extended our previously proposed model of lung infection with *Streptococcus pneumoniae* [5]. This model described dynamics of pneumococci population in lung and resulting innate immune response comprising neutrophils and macrophages in analogy to the model of Smith et al. [6], and additionally, the effects of antibiotic treatment. Due to lack of specific data, in our former model it was impossible to distinguish between alveolar macrophages and macrophages derived from invasive monocytes. IL-6 was considered as the major regulator of the immune response. No other cytokines or chemokines were considered.

We now improved this model by adding further important components of the immune response to make it biologically more plausible and to account for the refined data base available to us. First, we now distinguish between alveolar macrophages as a very early line of defense and the lately reacting monocyte-derived macrophages resulting in more realistic dynamics of the contributions of both lines of defense. We extended the network of cytokines and chemokines by adding IL-10 as a major immune-suppressive cytokine responsible for achieving steady-state values after successfully fighting the infection. We newly introduced a chemokine network to model migration of immune cells. In detail, we added CCL2, CXCL1 and CXCL5 which are major factors in murine immune response. Their biological functions are relatively well understood to allow mechanistic modelling [22, 33, 34]. To model treatment with NOX-D19, we put some emphasis on a refined model of barrier function and related extend of bacteremia, i.e. we modeled the transition of bacteria from alveolar tissue to blood stream in dependence on barrier function.

The resulting model is considerably more complex compared to the previous one. Calibrating this model was possible on the basis of an improved data base. Specifically, we now had access to much more closely meshed time points in the early phase after infection. While in the former modelling, we only relied on measurement points at 24h or later, we now had additional measurement points at 2h, 6h and 12h at our disposal. This early phase is characterized by rapid dynamics which are highly informative for model calibration. Moreover, improved experimental read-outs of the barrier function (i.e. serum to BALF albumin ratio and additional cytokines) allowed us to derive refined parameter estimates of the corresponding model compartments. Finally, TNF-*α* served as another outcome parameter of the model. Thus, experimental readouts of all state parameters of the model were available except for debris for which we used a semi-quantitative histologic score. As a consequence, most of the model parameters expressed good identifiability. However, there are also some issues regarding data quality: First, without antibiotics a few subjects were euthanized prematurely due to severe disease conditions, resulting in a survival bias affecting measurements at later time points. Second, low cytokine values typically showed larger measurement errors resulting in less precise data at the end of the measurement period for cured subjects. Finally, inter-individual variances in data are relatively high, covering orders of magnitude in a few occasions.

Besides modelling antibiotic treatment, we proposed a semi-mechanistic model of NOX-D19 action in mice for the first time by assuming a reduced damaging effect of invading immune cells on epithelium preventing barrier failure to some extent. Due to the different mechanisms of action of antibiotics and NOX-D19, we assumed independent effects. Accordingly, the drugs act synergistically but not additively due to the strong non-linearity of our model. It needs to be acknowledged that data under NOX-D19 treatment were much sparser, i.e. the early time points were not available for these experiments. Moreover, no data of combined NOX-D19 and antibiotic treatment were available. We used our model to predict the effects of such combination therapies offering a way to validate the model on the basis of future experiments.

Results of model simulations are in good agreement with the experimental data. However, parameters of the model are choosen in such a way that only two final states are possible (disease free and disseminated disease). States with a persistent residual infection are not covered. This might explain the slightly inferior agreement of model and data at late time points in the scenarios with antibiotic treatment.

A limitation of our model is that it is specifically developed for the situation of pneumonia induced by *Streptococcus pneumoniae*. Although this is by far the most frequent cause of the disease (up to 50% [35]) pneumonia may be caused by other bacterial and notably also non-bacterial pathogens such as viruses and fungi. It is not straightforward to generalize our model to these pneumonia pathogens, especially due to substantial differences in immune responses including expression of cytokine networks [36]. Modelling other pathogens would require other model structures as proposed in the literature (e.g. influenza: [37], fungal: [38, 39]). A model of pneumonia caused by *Acinetobacter baumannii* is presented by Diep et al. [40]. This model is based on rat data and describes bacterial population, neutrophils, albumin, and neutrophil-regulated cytokines and chemokines. Macrophages are not considered. Another model was proposed by Mochan et al. [41]. They considered dynamics of *Streptococcus pneumoniae* in lung and blood, neutrophil response and barrier damage but no cytokines or chemokines. Similarly, these players are also considered in the model of Domínguez-Hüttinger et al. [42], who modeled barrier failure in more detail by considering switch activating Toll-like receptors. Exponential growth and elimination of bacteria by the immune system in the blood vessels are included, and sepsis as well as immunological scarring are simulated. Although we considered bacteremia in our model, our model does not cover conditions of pneumogenic sepsis, i.e. a fulminant systemic immune reaction due to lung infection. This would require additional model extensions such as pathogen amplification and immune response in blood.

In conclusion, we proposed a comprehensive biomathematical model of *Streptococcus pneumoniae* including three lines of innate immune response, major cytokine effects, barrier function and effects of treatment including antibiotic therapy and adjunctive treatment with the experimental NOX-D19 spiegelmer therapy. The model was compared with a comprehensive set of experimental data with closely meshed time series in the early phase of infection, with or without treatment. We demonstrated utility of the model for making predictions regarding new therapy schedules. We plan to verify these predictions by future experiments and to extend the model by further therapeutic drugs and strategies.

## Supporting information

S1 File A biomathematical model of immune response and barrier function in mice with pneumococcal lung infection—Supplement material. The file contains estimated model parameters and corresponding sensitivity analyses.
(PDF)

S2 File A biomathematical model of immune response and barrier function in mice with pneumococcal lung infection—Data used for model development. The file contains observed values.
(TXT)
