# Supplementary material for: A biomathematical model of immune response and barrier function in mice with pneumococcal lung infection
Source: PLoS One. 2020 Dec 3;15(12):e0243147. doi: 10.1371/journal.pone.0243147 (PMC7714238; doi:10.1371/journal.pone.0243147)
Supplement: S1 File — The file contains estimated model parameters and corresponding sensitivity analyses. (PDF) [file pone.0243147.s001.pdf]

A biomathematical model of immune response and barrier function in mice with pneumococcal lung infection – supplement material

Sibylle Schirm[1]Peter Ahnert[1], Sarah Berger[2], Geraldine Nouailles[2], Sandra-Maria Wienhold[2], Holger Müller-Redetzky[2,3], Norbert Suttorp[3], Markus Loeffler[1], Martin Witzenrath[2,3], Markus Scholz[1,4*]

1 Institute for Medical Informatics, Statistics and Epidemiology, University of Leipzig, Leipzig, Germany
2 Division of Pulmonary Inflammation, Charité – Universitätsmedizin Berlin, corporate member of Freie Universität Berlin, Humboldt-Universität zu Berlin, and Berlin Institute of Health, Charitéplatz 1, 10117, Berlin, Germany
3 Department of Infectious Diseases and Respiratory Medicine, Charité – Universitätsmedizin Berlin, corporate member of Freie Universität Berlin, Humboldt-Universität zu Berlin, and Berlin Institute of Health, Berlin, Germany
4 LIFE Research Center of Civilization Diseases, University of Leipzig, Leipzig, Germany

* markus.scholz@imise.uni-leipzig.de

Parameter sensitivity

A comprehensive sensitivity analysis is performed to determine parameter identifiability. We calculated the deterioration in fitness function after changing the optimal value of a single parameter by $\pm 10\%$ while the other parameters were kept constant (see Figure S1). The parameters k_{P}, d_{CCL2}, k_{DEA}, $k_{\mathrm{CFUB\_P}}$, d_{IM}, k_{DN}, and $k_{\mathrm{N\_IL6}}$ are most sensitive.

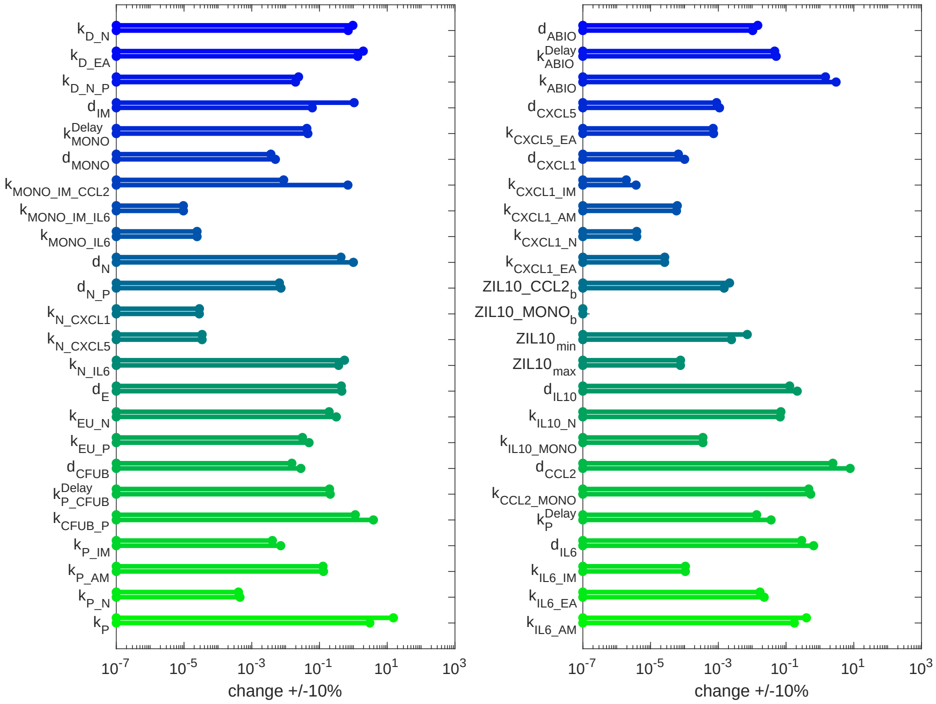

Fig S1. Parameter sensitivity 1. Single parameter values were changed by $\pm 10\%$ while the other parameters were kept constant. Corresponding relative deterioration of the fitness function was calculated as a measure of sensitivity of the considered parameter. Longer bars correspond to more sensitive parameters, i.e. better identifiability.

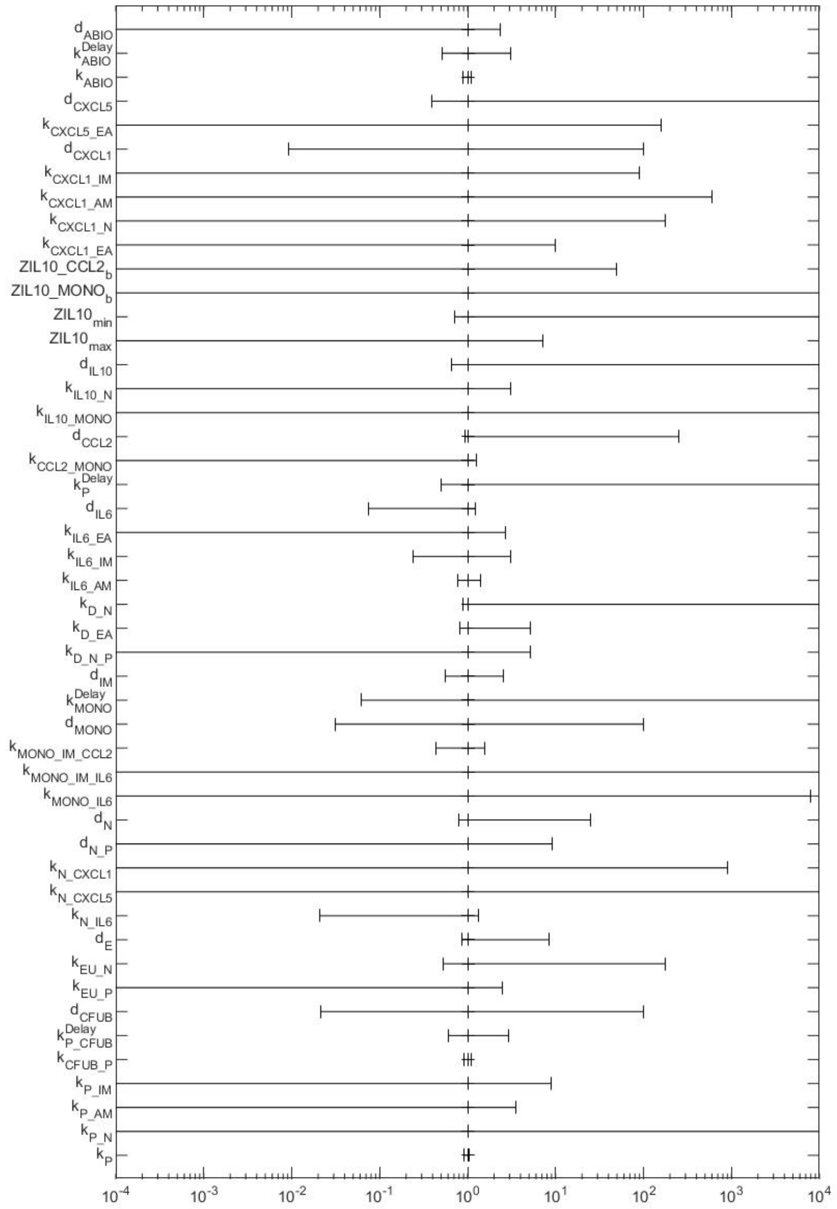

Fig S2. Parameter sensitivity 2. Single parameter values were changed until a deterioration of the fitness function of ±2.5% is reached. The other parameters were kept constant. The x-axis shows the corresponding relative change of the parameters. It revealed that for certain model parameters only upper or lower bounds are well identifiable.

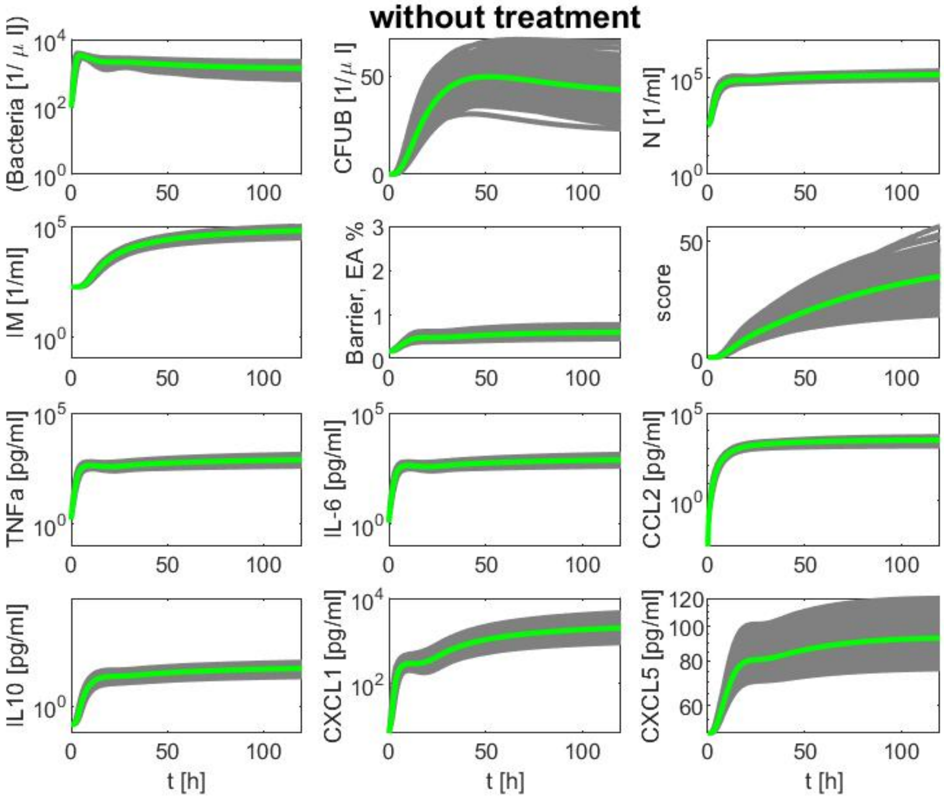

Fig S3. Certainty of prediction in a scenario without treatment. Parameter values were changed randomly with a variance of 0.1 (100 samples) and resulting model predictions are displayed in grey. The green curve corresponds to the parameter settings in Table S2, S3 and S4.

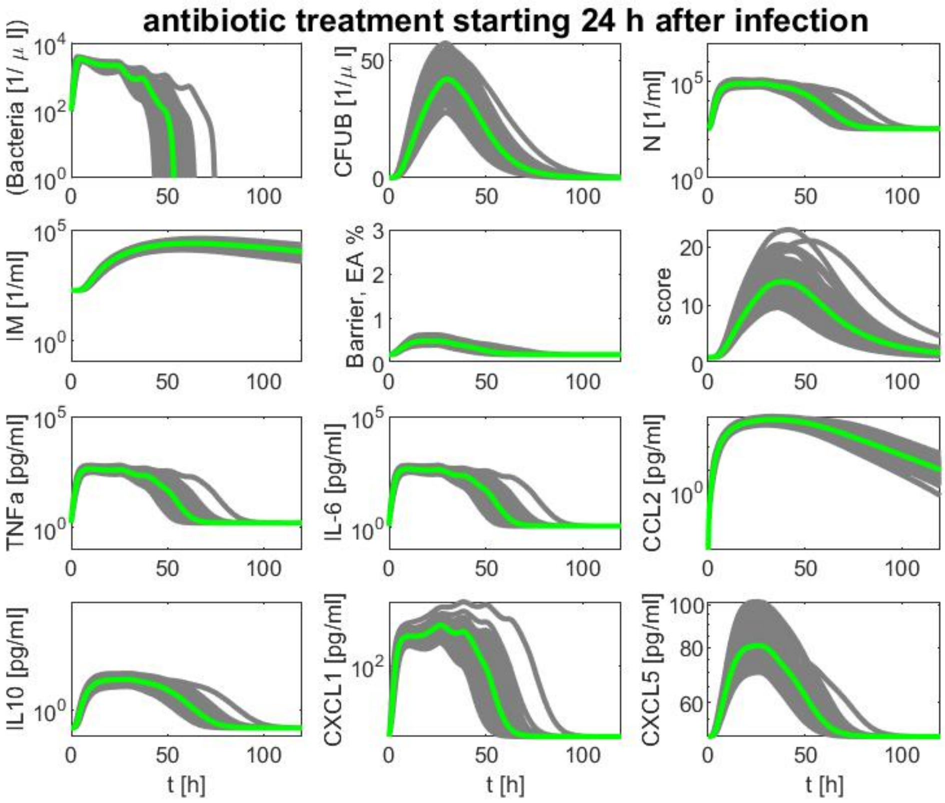

Fig S4. Certainty of prediction in a scenario with antibiotic treatment starting 24 hours after infection. Parameter values were changed randomly with a variance of 0.1 (100 samples) and resulting model predictions are displayed in grey. The green curve corresponds to the parameter settings in Table S2, S3 and S4.

Parameters

We here present initial conditions and parameter settings of our model.

Table S1. Initial conditions. Values were taken from Data.

P_0	bacteria in BALF	1.00E-06
$CFUB_0$	bacteria in blood	1.00E-06
EU_0	epithelial cells	9.98E+01
EA_0	activated epithelial cells	1.93E-01
N_0	neutrophils	3.51E+02
AM_0	alveolar macrophages	2.87E+04
IM_0	inflammatory macrophages	1.88E+02
$MONO_0$	monocytes	2.01E-01
$IL6_0$	IL-6	1.18E+00
$IL10_0$	IL-10	2.10E-01
$CCL2_0$	CCL2	0.00E+00
$TNFa_0$	TNFα	1.64E+00
IM_{sv}	condition for delay compartment (calculated)	1.07E-06
$CXCL1_0$	CXCL1	7.05E+00
$CXCL5_0$	CXCL5	5.02E+01
D_0	score	1.00E+00

Table S2. Cell kinetic parameters.

compartments *P* and *CFUB*			
EffInf	rate of colonizing bacteria	1.00E-03	set (data: 320-2560 /μl)
k_{P}	initial growth rate of bacteria	2.00E+00	fitted [1]
P_{max}	maximal number of bacteria	5.00E+03	set (data: 42-14200 /μl)
k_{PN}	bacterial clearance by neutrophils	2.45E-05	fitted
$k_{\mathrm{P\_AM}}$	bacterial clearance by AM	3.14E-04	fitted
$k_{\mathrm{P\_IM}}$	bacterial clearance by IM	4.10E-04	fitted
n	Michaelis-Menten constant	5.00E+00	set (not identifiable)
$k_{\mathrm{CFUB\_P}}$	migration rate of bacteria through barrier	2.30E+00	fitted
$k^{\mathrm{Delay}}_{\mathrm{P\_CFUB}}$	delay of bacterial migration	1.17E-01	fitted
d_{CFUB}	bacterial clearance in blood	4.72E+01	fitted
compartments *EU* and *EA*			
$k_{\mathrm{EU\_P}}$	epithelial cells activation rate due to bacteria	1.38E-07	fitted
$k_{\mathrm{EU\_N}}$	epithelial cells activation rate due to neutrophils	1.32E-08	fitted
P_{EU}	steady-state prodution of epithelial cells	4.64E-04	set (steady state cond.)
d_{E}	degradation rate of affected epithelial cells	4.19E-01	fitted
P_{EA}	steady-state production of affected epithelial cells	8.05E-02	set (steady state cond.)
compartment *N*			
$k_{\mathrm{N\_IL6}}$	neutrophil recruitment by IL-6	4.75E+01	fitted
$k_{\mathrm{N\_CXCL5}}$	neutrophil recruitment by CXCL5	6.66E-02	fitted
$k_{\mathrm{N\_CXCL1}}$	neutrophil recruitment CXCL1	1.50E-02	fitted
N_{max}	maximum number of neutrophils in alveolar space	1.59E+06	set (from data)
$d_{\mathrm{N\_P}}$	bacterial-induced neutrophil death rate	4.75E-06	fitted
d_{N}	neutrophil apoptosis rate	2.17E-01	fitted
P_{N}	neutrophil migration in steady-state	1.67E+01	set (steady state cond.)
compartments *MONO* and *IM*			
$k_{\mathrm{MONO\_IL6}}$	monocyte recruitment rate by IL-6	1.25E-04	fitted
$k_{\mathrm{MONO\_IM\_IL6}}$	monocyte differentiation rate by IL-6	1.02E-06	fitted
$k_{\mathrm{MONO\_IM\_CCL2}}$	monocyte differentiation rate by CCL2	3.09E-03	fitted
d_{MONO}	monocyte degradation rate	3.00E+02	fitted
P_{MONO}	steady-state influx of monocytes	6.04E+01	set (steady state cond.)
$k^{\mathrm{Delay}}_{\mathrm{MONO}}$	delay of monocyte differentiation	2.27E-01	fitted
k_{MONO}	translates units	1.00E+03	set
d_{IM}	macrophage degradation rate	2.15E-02	fitted
P_{IM}	steady-state influx of macrophage	4.05E+00	set (steady state cond.)
compartment *D*			
$k_{\mathrm{D\_N\_P}}$	debris from bacterial-induced neutrophil death	3.65E-10	fitted
$k_{\mathrm{D\_EA}}$	debris from epithelial cell death	1.93E-01	fitted
$k_{\mathrm{D\_N}}$	debris from neutrophil death	9.11E-06	fitted
d_{D}	debris degradation rate	4.05E-02	set (steady state cond.)

Table S3. Cytokine and chemokine related parameters.

compartment *IL-6*			
$k_{\mathrm{IL6\_AM}}$	IL-6 production by AM	1.97E-04	fitted
$k_{\mathrm{IL6\_IM}}$	IL-6 production by IM	2.30E-04	fitted
$k_{\mathrm{IL6\_EA}}$	IL-6 production by EA	1.65E+01	fitted
d_{IL6}	IL-6 degradation	3.61E+01	fitted
P_{IL6}	IL-6 production in steady-state	3.94E+01	set (steady state cond.)
$k_{\mathrm{P}}^{\mathrm{Delay}}$	delay of IL-6 production by macrophages	3.06E-01	fitted
compartment *TNFα*			
$k_{\mathrm{TNFa\_AM}}$	TNFα production by AM	1.97E-04	set (analog. to IL-6)
$k_{\mathrm{TNFa\_IM}}$	TNFα production by IM	2.30E-04	set (analog. to IL-6)
$k_{\mathrm{TNFa\_EA}}$	TNFα production by EA	1.65E+01	set (analog. to IL-6)
d_{TNFa}	TNFα degradation	3.61E+01	set (analog. to IL-6)
P_{TNFa}	TNFα production in steady-state	5.58E+01	set (steady state cond.)
compartment *CCL2*			
$k_{\mathrm{CCL2\_MONO}}$	CCL2 production by monocytes	5.12E+02	fitted
d_{CCL2}	CCL2 degradation	7.40E-02	fitted
compartment *IL-10*			
$k_{\mathrm{IL10\_MONO}}$	IL-10 production by monocytes	1.15E-01	fitted
$k_{\mathrm{IL10\_N}}$	IL-10 production by neutrophils	4.98E-05	fitted
d_{IL10}	IL-10 degradation	2.96E-01	fitted
P_{IL10}	IL-10 production in steady-state	2.17E-02	set (steady state cond.)
$ZIL10_{\mathrm{max}}$	maximum suppression by IL-10	1.38E-01	fitted
$ZIL10_{\mathrm{min}}$	minimum suppression by IL-10	1.39E+00	fitted
$ZIL10_{\mathrm{nor}}$	steady-state suppression by IL-10	1.00E+00	set (steady state cond.)
$ZIL10\_MONO_{\mathrm{b}}$	sensitivity of monocyte recruitment suppresion by IL-10	4.14E+00	fitted
$ZIL10\_CCL2_{\mathrm{b}}$	sensitivity of CCL2 production to IL-10	2.02E-02	fitted
compartment *CXCL1*			
$k_{\mathrm{CXCL1\_EA}}$	CXCL1 production by EA	1.65E+04	fitted
$k_{\mathrm{CXCL1\_N}}$	CXCL1 production by neutrophils	4.57E-02	fitted
$k_{\mathrm{CXCL1\_AM}}$	CXCL1 production by AM	1.68E-03	fitted
$k_{\mathrm{CXCL1\_IM}}$	CXCL1 production by IM	1.05E-02	fitted
d_{CXCL1}	CXCL1 degradation	4.90E+02	fitted
P_{CXCL1}	CXCL1 production in steady-state	2.58E+02	set (steady state cond.)
compartment *CXCL5*			
$k_{\mathrm{CXCL5\_EA}}$	CXCL5 production by EA	3.31E+01	fitted
d_{CXCL5}	CXCL5 degradation	3.28E-01	fitted
P_{CXCL5}	CXCL5 production in steady-state	1.01E+01	set (steady state cond.)

Table S4. Intervention related parameters.

t_{Pneu}	inhalation time	1.67E-02	set
t_{ABIO}	injection time for antibiotic treatment	1.00E-01	set
k_{ABIO}	antibiotic effect factor	6.92E+00	fitted
$k_{\mathrm{ABIO}}^{\mathrm{Delay}}$	delay of antibiotic effect	6.66E-01	fitted
d_{ABIO}	clearance of antibiotics	7.74E-03	fitted
$\mathit{ZD19}_{\mathrm{max}}$	maximal suppression by D19	0.00E+00	set
$\mathit{ZD19}_{\mathrm{min}}$	minimal suppression by D19	1.00E+00	set
$\mathit{ZD19}_{\mathrm{nor}}$	suppression for 1 mg/kg D19	5.84E-01	fitted
$\mathit{ZD19}_{\mathrm{b}}$	sensitivity parameter	3.27E-01	fitted

Model Predictions

Table S5. Prediction. Simulated maximum barrier impairment, maximum bacteremia and maximum pneumococcal population in BALF within the first 48 hours and the impact of antibiotic and D19 treatment thereon.

scenario	bacteria in BALF	bacteria in blood	barrier EA
without therapy	3.39E+03	4.96E+01	5.50E-01
2 mg/kg D19	3.59E+03	5.05E+01	4.12E-01
20 mg/kg D19	3.73E+03	4.58E+01	3.04E-01
antibiotics 24h	3.39E+03	4.18E+01	4.98E-01
antibiotics 24h, D19 20 mg/kg	3.73E+03	3.71E+01	2.94E-01
antibiotics 48h	3.39E+03	4.96E+01	5.50E-01
antibiotics 48h, D19 20 mg/kg	3.73E+03	4.58E+01	3.04E-01
relative change (with D19/without D19)			
antibiotics 24 h	1.10E+00	8.87E-01	5.91E-01
antibiotics 48 h	1.10E+00	9.24E-01	5.53E-01

References

1. Smith AM, McCullers JA, Adler FR. Mathematical Model of a Three-Stage Innate Immune Response to a Pneumococcal Lung Infection. J Theor Biol. 2011;276(1):106–116.
